# Supplementary material for: Motion of VAPB molecules reveals ER–mitochondria contact site subdomains
Source: Nature. 2024 Jan 24;626(7997):169–76. doi: 10.1038/s41586-023-06956-y (PMC10830423; doi:10.1038/s41586-023-06956-y)
Supplement: Supplementary file 1 — Supplementary Text, including sections 1–8—see Contents page for details. [file 41586_2023_6956_MOESM1_ESM.pdf]

nature portfolio

https://doi.org/10.1038/s41586-023-06956-y

Supplementary information

Motion of VAPB molecules reveals ER–mitochondria contact site subdomains

Supplementary Text

I. Technical limitations to studying contact sites between organelles 1
A. Sources of Identification artifacts 1
B. Sources of Perturbation artifacts 2
C. Sources and effects of averaging artifacts 2
D. A note on the role of this work in solving some of these problems 3
II. Additional literature and considerations 3
A. A note on nomenclature 3
B. Potential for subclasses of ERMCSs and implications 3
C. Contact sites with other cellular structures 4
D. A general note on the nature of dynamic processes in cell biology 5
E. Implications of the VAPB dimerization interface on the results in this paper 5
III. FIB-SEM and membrane reconstruction 6
A. A note on reasoning for FIB-SEM and manual reconstruction approach 6
B. A note on selection of potential sites of contact in FIB-SEM volumes 6
C. Method for smoothing and triangulation parameters 7
D. Method for 3D curvature analysis 7
E. Method for parallel and perpendicular curvature analysis and implications 7
IV. Molecular biology and plasmids 8
A. A note on construct choices and limitations of photophysics 8
B. Cloning strategy, construct design, and controls for tag perturbation 9
V. Imaging strategy and image preparation/processing 10
A. A note on imaging strategy, laser power, and image preparation 10
B. Imaging speed requirements for ER motion 11
C. Image filtering and preparation 11
VI. Single molecule tracking and trajectory analysis 11
A. A note on complications of tracking in arbitrary organelle geometries 11
B. Effects of organelle structure on linkage artifacts and trajectory formation 12
C. Effects of organelle structure on ensemble analysis of trajectories 12
D. Effects of organelle structure on single trajectory analysis and latent state determination 13
E. Use of Diffusivity Index to classify latent states 14
VII. Single contact site analysis and interaction probabilities 14
A. A note on probability distributions and coefficients used throughout this paper 14

B. Localization probabilities and localization density 15
C. Mitochondrial enrichment (ME) coefficient 15
D. Likelihood of trajectory binding 15
VIII. A note on reasoning for statistics, order of operations, and grouping of nested data 16

Supplementary Video 1. Reconstruction of membrane contact sites between the ER and the mitochondria using FIB-SEM. Segmentation and reconstruction from raw serial section electron microscopy volumes are shown for one of the two volumes used in the paper (Volume 1). Mitochondria membranes are colored blue, ER membrane is colored cyan, and regions of the ER membrane within tethering distance of VAPB are colored red. Note the alignment of the cristae with ERMCSs is generally pervasive in the volume.

Supplementary Video 2. Method of simultaneous imaging, localization, and tracking of single VAPB proteins in the ER. Realtime visualization of the location of the ER (cyan), Mitochondria (magenta), and single VAPB molecules (yellow) in the periphery of a representative COS7 cell (shown is one of 24 in this dataset). Resulting trajectories are shown at the end of the video. Note that most VAPB molecules are freely diffusing in the ER, making them available for new contact sites to form (see Ext. Data Fig. 3).

Supplementary Video 3. Single VAPB tether exchange between neighboring contact sites on a single mitochondrion. Movie shows individual VAPB molecular trajectories (slowed down 3-fold, for ease of visualization) color coded by molecule. Graphs on the left show distance of each molecule from the center of the associated contact site. Note the rapid exchange of some molecules between neighboring contact sites on the same organelle.

Supplemental Text

I. TECHNICAL LIMITATIONS TO STUDYING CONTACT SITES BETWEEN ORGANELLES

Contact sites between organelles have been extensively studied over the past forty years, primarily through reliance on traditional biochemistry and electron microscopy, though light microscopy has increasingly become an important validating tool in recent years[1,2]. These structures are particularly challenging to study, however, as they are generally very small, very dynamic, and very sensitive to experimental perturbation. We briefly describe here the risk of artifact associated with studying contact sites between organelles using existing technologies, which primarily fall into three categories. **Identification artifacts** are errors in the correct labeling of any potential organelle-organelle interaction as a specific contact site. **Perturbation artifacts** are errors in the measurement or conclusions drawn about contact sites as a result of the method of assaying them. **Averaging artifacts** are errors from the inherent loss of specificity that comes with pooling many independent samples together, either many cells with varied contact sites or even many diverse contact sites in a single cell. We detail sources of each of these errors we are aware of below.

A. Sources of Identification artifacts

1. Fixation—the number of apparent contact sites in EM data is significantly increased upon chemical fixation[3], so clear establishment of what was a contact site in the unfixed cell is challenging. This may partially be the result of dehydration during EM sample preparation and thus may be avoided in fixed light microscopy experiments, but since this has not been clearly demonstrated to be the case, live cell experiments are preferrable.
2. Nonspecificity of EM labeling—in the absence of correlative optical microscopy, it is challenging to know that just because two membranes are within a certain distance that a specific contact has been established. Deviations from the expected shape, electron density, or associated structures can be evidence of specific contact, but still cannot be easily associated with specific machinery. This is underscored by the data in this paper (e.g.- Extended Data Figs. 1b + 2d), where regions of known contact sites within tethering distance were shown to avoid membrane deformation that is expected for regions of high-density tethering.
3. Nonspecificity of colocalization approaches—Use of colocalization in diffraction-limited microscopy has become a popular tool for identifying contact site location in live cells[4–6]. Given the limited spatial resolution of light microscopy techniques, many locations in the cell will have two organelle compartments within a voxel of one another that may not be directly interacting. Some groups have addressed this by simply counting sites where contact-associated functions occur[4,7], but identification of *bona fide* contact sites where the desired function did not occur is then not possible. Others have required temporal associations[6], reasoning that two organelles should not stay in proximity unless directly tethered. This is probably a good strategy at the periphery of tissue culture cells, where there is lots of space for organelles to drift[8], but is likely to create many false positives in the dense perinuclear area where mechanical crowding may hold organelles together even when not specifically tethered.
4. Sampling bias from low coverage in EM—EM-based approaches convey very high spatial resolution, but this elevated information density comes with an obligatory loss in throughput. A number of studies have shown that single slices through a cell as are common in transmission EM studies are not necessarily good quantitative representations of the full cell volume[8–13], and the heterogeneity in contact sites described in this study (Extended Data Figs. 2+3) suggest tens of cells must be sampled

to capture the diversity of even a single tether at contact sites. Thus, this tool must be coupled to higher throughput approaches like biochemistry and fluorescence microscopy.

5. Incomplete penetrance of specific labels—The limitations of colocalization-based approaches have led many groups to shift to using more specific labeling technologies like FRET sensors[14,15], fluorescence complementation approaches[16–18], and dimerization dependent fluorescent proteins (ddFPs)[19]. These significantly increase the specificity of labeling, since no signal is generated unless the two membranes are in sufficiently close proximity for complementation to occur. However, it is unclear the proportion of contact sites these labels can access. Presumably, they can access contact sites where the membranes are close enough for the synthetic tethers to reach one another, but contact sites in some contexts have been described to be much larger than the "conventional" values of 10-30nm, even in excess of 50 nm[20,21]—these complementation approaches may not access these interfaces. Additionally, existing protein or lipid complexes at contact sites may affect how easily synthetic tethers can access the interface.

B. Sources of Perturbation artifacts

1. Fixation—Performing the throughput of EM required to quantitatively measure the effects of fixation on contact site size, structure, membrane spacing, or composition has not been possible, but it seems likely some or all of these things could be affected[3,22,23]. Future work will be needed to determine the scale of this, but cryo-based approaches, though laborious, may be needed to accurately extract the nanoscale structure of contact sites.
2. Specific label-induced expansion—Given the incredible fluidity of the ERMCS interface described in this work, it seems likely that they will be very sensitive to perturbation even by relatively innocuous experimental protocols. For instance, the measured affinity of ddFPs is in a similar range to that of VAPB for FFAT-motifs[24,25], and this is already a considerably lower stability interaction than complementation tools like more traditional spit fluorescent proteins that are commonly used. As we show in this work (Fig. 3), the size of the ER membrane necessitates that in most conditions the availability of the mitochondrial tether dominates the expansion or contraction of the contact site, and synthetic tethers by definition increase the availability of tethers in the mitochondria. Thus, these are likely to cause contact site expansion over time, even if the components are low affinity or of low temporal stability.
3. Specific label-induced protein displacement—Given our results with VAPB density in the central subdomain of the contact site, it is possible that this extreme enrichment of specific tethers in this region may have an effect on the ability of other contact site proteins to access this space. Although we have not directly examined this, it seems likely synthetic tethering pairs would also be capable of this effect.
4. Specific label-induced membrane spacing artifacts— As even the low affinity interactions of VAPB and PTPIP51 can cause significant expansion of contact sites at defined membrane spacing[26–28], it's possible that the low affinity synthetic tethers may also have this effect.

C. Sources and effects of averaging artifacts

Even when only using VAPB as a probe, cells likely have at least hundreds of contact sites between the ER and the mitochondria. If there are ERMCSs in these cells that VAPB does not enter or mediate, it is likely this number is a significant underestimate of the total number of actively-tethered interfaces. Our data suggests significant cell-to-cell heterogeneity in contact site behavior even in the relatively simple system of immortalized cells in culture. Approaches that pool many diverse biological sites like biochemistry and even flow cytometry

are likely to miss significant amounts of the subtlety in the system. Our data with P56S VAPB is a perfect example of this, where a very small fraction of the molecules are performing a medically-relevant behavior, but the vast majority of the molecules are not. This underscores the need for increasing the ease of experimental approaches with single biological event resolution.

D. A note on the role of this work in solving some of these problems

In this work, we have attempted to introduce tools and approaches that can be used to complement these existing experimental approaches in the literature. We do not yet know what proportion of contact sites detected by the means described above are mediated by VAPB, and it will be an exciting direction for future work to establish this and the role of other ER-mitochondria tethers at this interface. The sptPALM-based approach here provides an effective way to see all the contact sites where VAPB is actively engaging in tethering and can in principle be combined with tracking of other tethers to generate a more complete vision of the heterogeneity and distribution of contact sites in single cells. We show in this work that the size of VAPB-mediated ERMCSs is heavily regulated by the limiting mitochondrial component rather than VAPB itself (Fig. 3). As such, sptPALM of VAPB is likely less perturbative than the complementation-based reporters based on split FPs and FRET, since these obligatorily require expression of a mitochondrial component whose affinity is in the range of VAPB-PTPIP51 or higher[104]. However, future work will be needed to see if VAPB is able to label all sites identified by these reporters, since in principle they only depend on the distance between the membranes and not the presence of additional mitochondrial factors like PTPIP51.

II. ADDITIONAL LITERATURE AND CONSIDERATIONS

A. A note on nomenclature

Several converging lines of evidence from distinct fields over the years have established the sites of contact between ER and mitochondria as unique environments. This has resulted in several competing nomenclatures for similar or even identical things, based on the perspective of the investigators involved. For clarity, we briefly state here that we use the term ER-mitochondria contact sites (ERMCSs) to remain consistent with the literature published by other biologists studying the sties from the perspective of ER proteins like VAPB. We note that many in the field refer to the same sites as mitochondria-ER contact sites (MERCs), especially if their work is focused on the mitochondrial components of the interaction. We also point out that in the current literature, these are both distinct from the commonly-used term mitochondrial-associated membranes (MAMs) that refers to the distinct membrane fraction associated with mitochondria in a biochemical preparation. The MAM contains many contact site proteins, but exactly how the MAM is related to the ERMCSs/MERCs in living cells is still an area of great interest in the field.

B. Potential for subclasses of ERMCSs and implications

Increasing evidence in recent years suggests that there are multiple distinct subclasses of ERMCSs (reviewed here[20,29,30]). These have been proposed based on visible differences in structure from electron microscopy[20], direct readouts of various contact site functions[29], and diverse sets of tethers having distinct functional associations[30]. At this point, it is not yet clear which subclasses defined by any one of these mechanisms correspond to subclasses defined by the others, so it is difficult to globally estimate what proportion of contact sites a particular set of observations will be valid for. The selection of VAPB as our model tether is largely

motivated by its implication in many different ERMCS-associated functions, including lipid transfer and calcium signaling and regulation (reviewed here[31]), but there are some more recently appreciated and less well described ERMCSs for which VAPB is unlikely to be a contributing factor. As such, these ERMCSs will not be represented in the datasets within this study, since our approach as presented here will only identify ERMCSs where VAPB is undergoing interactions.

One example of this is the more recently described sites of contact between the mitochondria and ribosome-studded ER (i.e., rough-ERMCSs)[20,29,32–34]. These structures are generally believed to be characterized by much larger intermembrane distances (>50nm)[20], since ribosome access to intermembrane spaces significantly smaller than this is predicted to be sterically limited. This distance is significantly larger than the predicted tethering distance of VAPB and PTPIP51 (<30nm)[27], so it is unlikely VAPB would show specific interactions at these sites. Consequently, we have limited our analysis of FIB-SEM data to potential sites of contact that VAPB could plausibly mediate (see FIB-SEM section below), but in actuality we did not observe many rough-ERMCSs in the reconstructed volumes in this study. This may represent a lack of these structures in the cellular periphery where this work has been performed (these have been predicted to be RRBP1-dependent structures[34,35], and we have previously shown this protein is largely restricted to the perinuclear ER through binding to specific posttranslational modifications on microtubules[36]), or it could represent that the expanded rough-ERMCSs seen in previous work are structures specific to the specialized tissues and cell types where most of the literature has been performed (e.g. liver).

C. Contact sites with other cellular structures

In addition to ERMCSs, many other organelles show sites of specific contact with ER, and nearly all of these have several sets of known or proposed specific tethering molecules that facilitate their structure and functions[1]. VAPB itself is also known to interact with numerous other organelles other than mitochondria (including endosomes, lysosomes, peroxisomes, golgi, and plasma membrane) through specific binding partners that exhibit FFAT motifs (reviewed here[31,37]). We can see these contact sites in various forms in many cells as well (e.g., Fig. 1j, Extended Data Fig. 2), and the size of these contact sites is often quite distinctive from that seen at ER contact sites with mitochondria. In this work, we have focused on ERMCSs, but the approach we introduce here can in principle be applied to any other organelle pair for which a nonperturbative fluorescent label exists. That said, the current limitations of the technology are such that we can only see one other organelle at a time in any given cell.

The most well-characterized contact sites in mammalian cells are between the ER and the plasma membrane, which are accessible in the TIRF field of commercial microscopes and are generally arrayed nearly perpendicular to the imaging plane on account of the cells adhering to the glass coverslip. These contact sites share similarities with and have differences from ERMCSs. Like ERMCSs, ER-PM contact sites can respond to cellular stimuli over rapid time scales[38,39], though admittedly nearly an order of magnitude slower than the time scales suggested for ERMCSs[6]. Classic early adaptations of sptPALM at the plasma membrane analyzed the motion of STIM1 and its binding partner Orai1[40] suggested dwell times measured in tens or hundreds of seconds, presumably through the interactions with cytoskeleton-supported domains in the plasma membrane. By contrast, dwell times for VAPB at ERMCSs were ~500 msec.

VAPB (and its closely related homolog VAPA) both directly interact with a number of proteins at the ER-plasma membrane junction (reviewed here[37]). Although sptPALM of the VAP proteins has not been performed at this site to our knowledge, there is data showing dynamic interactions of the binding partners[41], and direct photoactivation release experiments of VAPA have estimated the dwell time of VAP proteins at these interfaces to be significantly longer than we observe with ERMCSs[42]. Interestingly, even at these slower exchange rates, the authors suggested this as a potential mechanism for phosphorylation-regulation of tethering components[42].

ER contacts with the plasma membrane are easily visible as enrichments with diffraction-limited microscopy[38–44], suggesting tether interactions at ERMCSs are far less stable than at ER-PM contacts. One stabilizing factor at the PM could be the cortical actin cytoskeleton[45], which is significantly larger and more complex than the actin associated with mitochondria[46–48]. Indeed, interactions of PM proteins with this actin cortical meshwork have been shown to have dramatic effects on plasma membrane protein diffusion through restriction of lateral diffusion[49–51].

D. A general note on the nature of dynamic processes in cell biology

The advent of live cell imaging has revolutionized the ability of cell biologists to study dynamic processes, but it has also revealed a remarkable range of time scales over which biological phenomena can occur. Many systems show relevant behavior on many different time scales. We have previously shown contact sites to be dynamic structures that can form or disengage on time scales of tens of seconds[6]. In this work, we have shown that the machinery that mediates these interfaces is significantly more dynamic, entering and exiting the sites themselves on time scales measured in milliseconds. To our knowledge, this speed of exchange is relatively unprecedented for membrane interfaces, since comparable work with very similar tethering proteins (VAPA) at the plasma membrane (see Supplementary Text, Section 2c above)[42] is predicted to be much more stable based on photoactivation release experiments. This likely explains the historic difficulty in labeling ERMCSs in traditional fluorescence microscopy assays, since fluorescent molecules do not enrich as dramatically as seen at ER-plasma membrane contacts, which can be visualized more clearly with diffraction-limited microscopy.

E. Implications of the VAPB dimerization interface on the results in this paper

VAPB has a well characterized dimerization interface, primarily mediated by the coiled-coil domain (aa 159-196)[31,37]. In this work, we do not uncouple the contributions of the dimerization capacity of VAPB from its direct PTPIP51-binding functions, both of which may contribute to the ERMCS-localization and function but are mediated through physically distinct domains. To ensure lateral interactions do not confound our experiments, the delta-N-term-VAPB construct is deliberately chosen to remove both the coiled-coil domain (aa 159-196) and the MSP domain (aa 7-124)[31,37]. Future work will be needed to uncouple the contributions of these two domains, since regulation of the two domains may present distinct phenotypes in terms of ERMCS regulation or function. This is of particular interest with regards to our results with the P56S mutant VAPB showing altered contact site dynamics. Since it remains contentious whether P56S VAPB can directly engage in binding with PTPIP51[52–54] and the disease always manifests in patients with a heterozygous genetic makeup (reviewed here[55,56]), it is possible that the phenotype observed also requires the unlabeled endogenous VAPB (or VAPA) that is present in the cell. It is at this point unclear whether WT VAPB interactions with P56S VAPB are entirely mediated through the coiled-coil dimerization domain or if improperly folded MSP domain association can also feed this interaction (reviewed here[31]). Either way, the trapped P56S VAPB domains are still likely to be mediating VAPB-associated functions or signaling

either directly or through the WT VAPB they recruit, possibly causing aberrantly long-lived signaling domains. This is supported by their slight but significant increases in size (Extended Data Fig. 9c) and slowed average diffusion landscape (Extended Data Fig. 9d), compared to normal ERMCSs. Thus, they remain a subject of great interest in future work to uncover specific mechanisms of disease.

III. FIB-SEM AND MEMBRANE RECONSTRUCTION

A. A note on reasoning for FIB-SEM and manual reconstruction approach

High pressure freezing followed by freeze substitution and FIB-SEM has been successfully used to understand many of the structural aspects of organelle membranes in the absence of fixation-induced artifacts[3,8,10,23,57], but the warping and staining effects of freeze substitution are still not well understood or mapped[23]. In principle, approaches performed in vitreous ice like cryo-ET, cryo-FIB-SEM, and cryo-EM avoid this issue, but in our hands these approaches do not offer sufficient contrast on ER membranes to perform nanoscale curvature or structural analysis. Additionally, the small volumes possible in these techniques make it hard to ensure that a ERMCS can be found within any given volume examined. As these technologies develop further, it will be exciting to examine this more closely, but current data suggests that warping effects in freeze substitution seem to effect local environments similarly, so the effects tend to be at the micron scale rather than the nanometer scale[23]. Thus, our local curvature analysis of the membrane is probably the best currently possible, but we will look forward to more careful analysis as cryo-based technologies for looking at membranes improve.

The most complete analyses of ER and mitochondria shape to date were also performed on high pressure frozen and freeze substituted FIB-SEM, but both used automated machine learning-based approaches for identifying the location and shape of membranes[8,21]. Although these provided a good sense of global ER and mitochondria shapes, we found that implementation of these types of approaches in our own data led to many errors in voxel classification in the tight space where the two opposing organelle membranes are in close proximity. Although these errors were often only a single voxel, they had dramatic effects on the curvature for the resulting triangulated surfaces, inhibiting our ability to analyze nanoscale membrane curvature in the contact sites themselves. Thus, we opted for a much smaller sample size and the somewhat laborious method of manual annotation of the data, but future work will examine the potential of training automated networks to minimize these types of artifacts.

B. A note on selection of potential sites of contact in FIB-SEM volumes

Studies in multiple diverse cell and tissue types and under various conditions have established a large range of observed intermembrane distances for ERMCSs (see Supplementary Text, Section 2b, above)[14,18,32–34,58,59]. The extent to which these represent functionally distinct structures as opposed to a single or a few structures dynamically changing distances to regulate function remains an area of exciting work in the field[20]. At this point, it is not clear which subset or subsets of these categories VAPB or any other specific tether is involved in, so we sought to be inclusive of any sites where it could potentially function as a tether. In cells overexpressing PTPIP51 and VAPB, where this tethering pair is likely to be the dominating factor in controlling the intermembrane distance, we find the mean intermembrane distance to be in the range of 20nm, in agreement with previous work[27]. Consequently, we considered an ERMCS to be any place that the ER and the mitochondrial outer membrane came within 24 nm (using a multiple of 8nm, our voxel size, decreases the likelihood of smoothing artifacts perturbing the edges of the potential contact sites). If the longest dimension of the putative ERMCS in the ER membrane was less than 30 nm, we discarded the site as a site of

coincidental proximity (these sites never showed altered curvature compared to the surrounding ER, see Supplemental Text, Sections 3d-e and Extended Data Fig. 7).

C. Method for smoothing and triangulation parameters

The voxels in our FIB-SEM data are approximately 8nm in all dimensions, which is not sufficiently far from the scale of membrane curvature to avoid significant contributions to local curvature analysis. In order to minimize this source of error, we reasoned that we could use *a priori* information about the shape of the organelle membranes themselves. Significant effort in the field has been contributed to understanding the local shape of membranes, both at the level of high-resolution transmission EM and through modeling and prediction from *in vitro*-derived biophysical parameters. Thus, we chose smoothing parameters for each organelle surface that are selected to create a curvature that is most close to the established literature values. These parameters are implemented blindly to all the data in our volumes to avoid bias. All smoothing and triangulation steps were performed as an automated analysis pipeline in Amira that is available upon request.

D. Method for 3D curvature analysis

Once triangulated surfaces were generated for the ER, we calculated the local curvature by fitting a quadratic form to the surface at each triangle using a 20-triangle neighborhood (approximately 25-30 nm, in our data). This process was iterated five times to smooth potential voxelation artifacts. Diagonalization of the resulting Hessian matrix produces eigenvectors and corresponding eigenvalues (C_1 and C_2) that represent the principal curvature axes of the surface at this resolution, and we calculated the resulting mean curvature value directly at each triangle as:

$$Mean\ Curvature\ = \frac{C_1 + C_2}{2}$$

Thus, convex regions have positive curvature, concave regions have negative curvature, and saddle points have net zero curvature. Note this is different from mean Gaussian curvature that is often used in the literature, where saddle points would be defined as negative curvature and both convex and concave structures have positive Gaussian curvature. Comparison between ERMCS curvature and non-ERMCS ER curvature was done by simply classifying the triangles as ERMCS-associated or not as described in the Section 3b above. Of note, all of the putative ERMCSs in this study showed net negative curvature by this method except two (of 38), which are directly proximal to one another and shown in Extended Data Fig. 1b. This is quite distinctive from the non-ERMCS ER membrane, which is uniformly positively curved (Extended Data Fig. 7e-f). Additionally, we point out that the units for this curvature are arbitrary, since the size of the triangles in our triangulated surface is not constrained to allow the most accurate fit (see Supplementary Text, Section 3c above), so we have represented them normalized to the median curvature value in the contact site, which was the most uniform number collected in the dataset (Extended Data Fig. 7f), presumably since mitochondrial shape is much more uniform than that of isolated ER (Extended Data Fig. 7d) .

E. Method for parallel and perpendicular curvature analysis and implications

The method used above for evaluating curvature provides a reasonable qualitative measure for local curvature at the nanometer scale that single proteins may experience, but it generally does not provide good micron scale predictions of the global structure or the extent to which membranes are deformed across a several hundred nanometer contact site. Consequently, we implemented a second, independent approach to address the changes in the ER shape at ERMCSs. Rather than allowing the Hessian matrix diagonalization to select the local

principal curvature axes for us, we manually aligned the axes to be parallel or perpendicular to the central axis of the dominating cylindrical structure in the proximity of the ER membrane (Mitochondria at ERMCSs, the longest local ER tubule in the case of isolated ER). The isotropic nature of FIB-SEM allows us to generate 2D EM slices along any arbitrary axis, and so we performed this manually by re-slicing along these axes at each structure using Fiji. The resulting 2D EM images (e.g., Extended Data Fig. 7a) present a representative cut along the micron-scale structure, and they provided a medium to manually trace the structure of the ER membrane. The resulting trace was fit to a circle using an MLE method. Comparison of the best fit radius of curvature (Extended Data Fig. 7b) showed that the radius was significantly larger for ERMCSs than for matched ER controls taken from ER structures within 1 micron of each ERMCS (Extended Data Fig. 7c), since the radius of mitochondria is significantly larger than the radius of the average ER structure. Structures where the center of the best-fit circle was inside the ER were classified as positive curvature, those outside the ER were classified as negative curvature (Extended Data Fig. 7b). Structures whose fit failed or produced a radius curvature > 1 micron represent ER membrane with neutral curvature, and these measurements were always parallel to the dominating cylindrical axis. Performing this for each of the 38 ERMCSs in the dataset and matched controls of ER in the surrounding area, we compared the curvatures along each axis defined as

$$\kappa = \frac{1}{R_{curvature}}$$

where κ is measured in inverse microns. These were plotted against one another, showing a few distinct types of ERMCS structures in the dataset (Extended Data Fig. 7d). In this representation, the predominantly tubular structure of ER membrane outside of the ERMCS clusters along the y axis of the plot, since these are universally highly positively curved perpendicular to the tube axis and show nearly neutral curvature along the tubule length, excepting where pearling instabilities create swellings in the tubule (reviewed here[60]). ERMCSs, however, show uniformly negative curvature along one axis if they touch the sides of mitochondria and negative curvature along both axes if they touch the tips (Extended Data Fig. 7d). Note the exception in the lower right quadrant of the plot is the paired contact sites shown in Extended Data Fig. S1b and discussed above, which are associated with a putative mitochondrial division site, and maintain positive curvature in the parallel dimension as the mitochondrion compresses around them. The two ERMCSs in the upper left quadrant of the plot are the smallest two ERMCSs in the dataset (~40nm across), making fit along their smaller dimension very imprecise; but we note that the fit along their longer dimension correctly identifies them as being associated with the side (neutral curvature) or the tip (negative curvature) of the contact site.

IV. MOLECULAR BIOLOGY AND PLASMIDS

A. A note on construct choices and limitations of photophysics

Performing sptPALM in the ER requires an ER counterstain to inform the correct localization linkages that are needed to generate trajectories. We found that counterstains targeted to the membrane like mEmerald-Sec61b were good labels, but at high expression levels they showed a detectable effect on the diffusive properties of the single molecule tracers in the membrane, presumably as the result of molecular crowding. Thus, we elected to use an ensemble marker for the ER (PrSS-mEmerald-KDEL) that resides entirely in the lumen to avoid perturbing tether motion.

Since VAPB also tethers FFAT-containing proteins on a number of non-mitochondrial membranes (reviewed here[31,37]), we required two labels (one for ER and one for mitochondria) in order to identify the contact sites that were associated with mitochondria (see Fig. 1j and Extended Data Fig. 2). Although we could not directly visualize the mitochondrial tether and test its sensitivity to OMM labeling, we decided to target our mitochondrial tether to the mitochondrial matrix to avoid this potential pitfall.

The selection of two ensemble fluorescent labels that must be run simultaneously with sptPALM is challenging, since labels that are either too far red shifted or blue shifted can perturb the experiment. Fluorescence that is too red-shifted can create false localization or decrease true localization precision by elevating the background, and fluorochromes in this excitation profile are susceptible to bleaching by the 647nm laser used for single molecule localization. Fluorescent labels that require significant blue-shifted excitation light will cause too much photoconversion of PA-JF646 and lead to localizations that are too dense to unequivocally track. Unfortunately, in many cases these complications are not obvious during data acquisition, and only become clear after a laborious processing pipeline. To minimize the wasted effort this entails, we selected the two ensemble markers used throughout this paper that gave us the largest proportion of usable data (PrSS-mEmerald-KDEL and mitoRFP), though these two markers can induce ER stress at late time points. As a result, all data presented in this paper is collected at less than 24 hours post transfection, when ER morphology and function were deemed to still be reasonably healthy (and the motion of single molecule tracers was consistent with earlier time points).

B. Cloning strategy, construct design, and controls for tag perturbation

The ER ensemble marker (PrSS-mEmerald-KDEL) was generated because in our hands mEmerald showed the best balance between the required brightness, obligate photostability, and toxicity effects of overexpression of green fluorophores. Briefly, PrSS-mEmerald-KDEL was generated by replacing the mRFP cassette in ER-mRFP using NEBuilder to exchange the catalytic cores of the fluorescent protein with homologous arms. The plasmid was sequence verified before use, and the plasmid sequence and map are available at Addgene or by request.

HaloTag-TA was generated by PCR amplifying a codon optimized version of HaloTag[61,62] and adding flanking AgeI and BsrG1 restriction sites using the primers in Table S1. The resulting product was inserted into mEmerald-Sec61b-C1 by digesting both constructs with AgeI and BsrG1 and ligating the purified products. The plasmid was sequence verified before use and the map and sequence are available at Addgene or by request.

All other single molecule tracer constructs were made from HaloTag-N1 and HaloTag-C1 backbones, which were generated by replacing the core of EGFP-N1 and EGFP-C1 with the same HaloTag core as used for HaloTag-TA above. This was performed using NEBuilder and the primers described in table S1 according to the manufacturer's recommendations. Note the very C and N terminus of EGFP were retained when fused to the linker sequence. This avoided folding issues and increased linker flexibility. The GFP-tagged versions of the proteins used in this paper have been previously shown to be functional[63].

To ensure that the HaloTag-linked versions of VAPB still retained their contact site-associated interactions, we generated both N- and C- terminally linked versions of VAPB by inserting the full length VAPB (amino acids 1-243) into both HaloTag-N1 and HaloTag-C1, using NEBuilder. The resulting constructs were sequence verified and then checked for behavior with sptPALM. Both constructs showed clear interactions at the interface of ER and

mitochondria, but a significant number of the C-terminally tagged VAPB molecules were freely diffusing in three dimensions within the cytoplasm. We presumed this represented a defect in the tail anchor insertion pathway as a result of adding the bulky HaloTag to the C-terminus, which must be passed through the membrane and face the ER lumen. As a result, we performed the remainder of the experiments in the paper using the N-terminally tagged versions of VAPB. The maps and plasmid sequences are available at Addgene or by request.

The VAPB construct with the N-terminus deleted was generated by inserting the last 32 amino acids of VAPB (amino acids 212-243) into HaloTag-C1 using NEBuilder. This deletion removes both the PTPIP51-interacting domain and the dimerization domain, essentially leaving only the transmembrane tail anchor and short sequence on either side. This proved to be sufficient for targeting to the ER, but it did not convey any detectable specificity to the motion of the tethers in the contact sites themselves (Fig. 1k-m), suggesting that VAPB does not use it's TM domain to sense the unique lipid environments of the contact site. An annotated map and plasmid sequence are available at Addgene or by request.

The VAPB-N-terminus fused to the tail anchor control from Sec61b was generated by PCR amplifying the synthetic HaloTag-VAPB fusion from HaloTag-VAPB, but only amplifying the N-terminal region (amino acids 1-211). This construct was inserted in place of mEmerald in mEmerald-Sec61b-C1 using NEBuilder. The resulting construct was sequence verified before use, and the map and sequence are available at Addgene or by request. This construct was also entirely targeted to the ER membrane, but it did show distinct regions of interaction at the mitochondria-associated contact sites (Fig. 1k-m).

HA-PTPIP51 has been previously shown to retain its function[28], but the protein's ability to tolerate a larger tag was unclear. However, we wished to perform experiments in live cells, where the HA-tag was not a reasonable labeling option. In order to surmount this, we extracted the full length PTPIP51 (amino acids 1-470) from HA-PTPIP51 using the primers in Table S1 and fused it directly to the modified EMCV IRES sequence from pHAGE-Tet-STEMCCA and mTagBFP2 from mTagBFP2-N1 using NEBuilder. The full sequence was inserted into EGFP-N1 replacing the EGFP. The resulting plasmid map and sequence are available at Addgene or by request. The construct was sequence verified before use and allowed the overexpression of PTPIP51 without any additional tags, while simultaneously producing mTagBFP2 in the cytosol to mark the transfected cells.

V. IMAGING STRATEGY AND IMAGE PREPARATION/PROCESSING

A. A note on imaging strategy, laser power, and image preparation

One of the most challenging aspects of sptPALM with multiple simultaneously-collected ensemble labels is that green labels that are often preferred for imaging require 488nm light for excitation. 488nm light can inefficiently cause photoactivation of the PA-JF646 dye used for sptPALM in our experiments. We tested a number of conditions and constructs, but found that even without any 405 radiation to photoconvert the dye, nonspecific photoactivation by the 488nm laser became problematic for us if greater than 100μW was applied to the back aperture (this corresponds to only approximately 272 mW/cm^2). Thus, if cells could not be visualized beneath this power, they were not able to be used for ER-based sptPALM. This is generally not enough excitation light to visualize even the brightest green ER structural labels when imaging camera exposure times are less than 20 msec, so we required some image processing steps to improve the signal to noise in the structural channels, described below.

B. Imaging speed requirements for ER motion

We have previously shown that even at 95Hz some molecular motion of membrane proteins is under sampled[64], and localization distortion from molecular motion is present even at this speed[65]. Keeping molecular steps small enough to identify single ER junction transitions requires a minimum of 15-20Hz in the peripheral ER[64]. However, the ER structure itself does not require such fast imaging to track unequivocally. Several papers throughout the years have quantified the high-speed motion of the peripheral ER using fluorescent labels for the structure[9,66–76]. Major structural rearrangements like tubule extensions[66–68], ring closures[77], and three-way junction formation[78–82] occur on a time scale of hundreds of milliseconds to seconds. We and others have described very high frequency oscillations in ER tubules that occur in excess of 40-50Hz as a result of cytoplasmic fluctuations[9,76], but these are often subdiffraction-limited in size and occur perpendicular to the central axis of ER tubules (and as such do not confound single molecule linkages along the tubules). Thus, we reasoned that if our effective imaging speed for the ER structure was in the range of 10Hz, we should still achieve Nyquist sampling for structural motion even if the structure was not imaged as quickly as the single molecule localizations required.

C. Image filtering and preparation

To accomplish this, the ER and mitochondrial channels were subjected to a 10-frame median filter in the time domain before being utilized for subsequent tracking support (effective exposure time, 110msec). The single molecule channel was left unprocessed, to avoid distorting the localizations or confusing the trajectories. The resulting filtered images still occasionally showed signs of bleaching in one or both channels, so we also performed a simple ratio bleach correction before subsequent image processing. The temporal median-filtered and bleach corrected image data is used for all ER and mitochondria images in this paper.

The curation of trajectories that is required to avoid linkage artifacts in sptPALM of ER proteins is a relatively laborious process as it was performed in this study. In order to make this as simple as possible, we performed a number of additional steps to help the trajectory curation. First, we generated a very crude mask of the ER using a simple threshold to generate a binary image and then performed a single-pixel dilation three times sequentially. Since the pixels are very large (160nm), this functionally expanded the mask to include all regions of the cell that contained ER. Localizations that fell outside this mask we removed before performing tracking. These events were rare, likely representing unbound dye, but in practice they can be distracting while setting tracking parameters.

VI. SINGLE MOLECULE TRACKING AND TRAJECTORY ANALYSIS

A. A note on complications of tracking in arbitrary organelle geometries

Single particle tracking (SPT) has proven to be a powerful tool for the analysis of biological phenomena at the molecular level in diverse environments[83–85]. Due to the high precision nature of the technique, nearly every step in the experiment and analysis pipeline from localization to tracking to analysis makes relatively heavy use of mathematical approaches. It is important to note that most of these approaches make some inherent assumptions about the boundary conditions and symmetry of the system. When tracking in contexts where symmetry is reasonably uniform (such as the ventral plasma membrane of a cell cultured on glass) these assumptions introduce minimal error into the analysis. However, when localizing and tracking molecules in the environment of morphologically complex cytoplasmic organelles, these assumptions can lead to significant artifacts. In this section, we briefly

discuss sources of error and controls that can be run to minimize or account for these errors, which we have performed throughout this study.

B. Effects of organelle structure on linkage artifacts and trajectory formation

Most traditional linking algorithms for SPT use an initial linking step based on nearest neighbor analysis that is then subjected to a subsequent optimization function[83,86]. In the context of an arbitrarily shaped organelle like the ER or the mitochondria, the nearest localization in 2D space is often not the nearest neighbor in the space in which the particles reside (and in the case of the mitochondria is often not even in the same organelle). We found by manual inspection of the data that even at modest linking densities the majority of trajectories showed steps that crossed at best prohibitively large distances through the structure, if not transitions that should be altogether forbidden. Future work may develop automated algorithms to use the underlying structure to directly inform the linking process, but in this study, we performed this step manually. The experimentalist was blinded to the condition and went through each frame of the time lapse manually breaking trajectory linkages that stepped over the polygonal spaces of the ER, which would have required a molecule to travel much more quickly through a circuitous route than is reasonable. As a qualitative validation, this process was performed on datasets collected in similar ER structures at a range of photoconversion densities, and subsequent work was performed at densities where the linkage properties were insensitive to localization density.

C. Effects of organelle structure on ensemble analysis of trajectories

Most statistical approaches for analyzing single particle trajectories make some base assumptions about the symmetry or at least isotropy of the system, especially the contribution of thermal fluctuations and Brownian motion[83]. Molecules confined to organelles without a high degree of symmetry are not necessarily subject to these assumptions, since diffusion can make significant contributions to apparently “directional” motion if some dimensions are more constrained than others. We have previously shown that both traditional mean-squared displacement (MSD) analysis and velocity autocorrelation produce erroneous results in the endoplasmic reticulum if the organelle shape is not accounted for[64]. A corrective approach can be used effectively in ER tubules, where *a priori* knowledge of the membrane shape allows the effective reduction to a one-dimensional problem, but without an orthogonal method of assaying contact site structure at this resolution it cannot be used to understand particle motion in the contact site. Thus, in this study we utilized a simple approach to minimize the error rather than try to remove it completely.

Briefly, we reasoned that since molecular diffusion operates over much faster time scales than organelle restructuring, in the limit of vanishingly small subregions of the ERMCS, most molecules within a reasonable time window should see approximately the same structure (Extended Data Fig. 6). As a result, we make the assumption that they should experience approximately similar diffusion and energetic environments, treating them as a constants and solving the overdamped limit of the Langevin equation for the trajectories in each neighborhood:

$$\frac{d\vec{r}}{dt} = \frac{\vec{F}(\vec{r})}{\gamma(\vec{r})} + \sqrt{2D(\vec{r})}\xi(t)$$

where the left term is a generic “drift” term that is left to account for non-diffusive aspects of the motion, and the right term estimates the Brownian motion in the neighborhood[86]. Additionally, the contribution of the “drift” term used to solve the equation is deliberately

unconstrained to avoid bias about the nature of contact site binding, and in this region where confinement to the site is significant it does contribute. However, this contribution serves to decrease the signal to noise for changes in D_{eff} across the contact site, and as such biases against our conclusions of subdomain-specific variation. This approach of breaking diffusion landscapes down into smaller subdomains has been used effectively in a variety of biological structures[87–89], though we caution that this does not completely remove the effects of structural variation, it simply reduces them. As a result, the numbers and D_{eff} extracted using this technique are not directly comparable to those collected using the more precise approaches based on single trajectories detailed below or our previously published work[64], which are in close agreement. However, this approach has served as an effective way to map the landscape of contact sites qualitatively, since molecules within the same contact site are likely subject to similar environments and analysis of hundreds of contact sites provided very consistent results.

One weakness of this approach, however, is that it not very effective in parsing changing state behavior, especially when the states are not uniformly changing as a function of location. Thus, in healthy ERMCSs, molecular behavior is more or less consistent within each neighborhood and low D_{eff} regions correspond well to where molecules are more likely to be located (Fig. 2k, Extended Data Fig. 6). However, in the P56S VAPB tracking, subdomains that trap P56S VAPB can often also have normally diffusing molecules present. Thus, these present as domains of very low diffusion using this approach, but future work will be needed to ascertain what proportion of this motion is truly "slowed diffusion" as opposed to increasing confinement as a result of lateral aggregation to other VAPB molecules or more stable binding to PTPIP51. There is some evidence for both of these as potential options[28,52,90,91] (despite ensemble biochemistry suggesting a loss of P56S interaction with PTPIP51[52,53,92,93], probably the result of the majority being in ER-localized aggregates[94]). However, parsing these states will require a more complex approach (see below) that is beyond the capacity of our current technology.

D. Effects of organelle structure on single trajectory analysis and latent state determination

Ensemble-averaged approaches like MSD or velocity autocorrelation have significant limitations in real biological systems in that they by necessity have trouble with temporally or spatially varying forces or contributions to motion[95]. In a dynamic and complex organelle structure like the ER or mitochondria, both spatial and temporal variation in the organelle's shape have significant contributions to molecular motion[64]. Approaches like traditional Hidden Markov Models can surmount this problem, but they require two significant constraints that are in practice nearly impossible to achieve with such asymmetric boundary conditions[96–98]. First, trajectories must be long enough to allow statistical convergence of the model, which can in practice be challenging to achieve with single molecule probes due to natural bleaching of the fluorochrome under the requisite levels of radiation. Second, they require an accurate upper bound on the number of states possible in the system. In practice, this requirement is essentially impossible to achieve in the highly complex and variable structure of the ER, since variations on local curvature, topology, connectivity, and protein/lipid content can produce many functionally distinct states. In this work, we take advantage of the superior photostability of new photoconvertible fluorochromes[99–101] to address the first point, and a model-independent way of determining latent states to address the second[102,103].

The complete approach used in this work is detailed elsewhere[102,103], but in brief the trajectories with sufficient length were broken into segments where behavior was deemed similar using a nonparametric Bayesian approach. In practice, the resulting segments were

often broken such that they correctly identified structural differences in the underlying ER (Extended Data Fig.4a), suggesting the tool is able to effectively identify the underlying structure. For each segment of a trajectory that was long enough (generally >150 steps), an implementation of the two-dimensional overdamped Langevin equation was solved:

$$d\vec{\boldsymbol{r}}_t = \gamma^{-1}\vec{\boldsymbol{F}}(\vec{r}_t)dt + \sqrt{2D}d\vec{\boldsymbol{B}}_t$$

(Note that although the model and lettering notation are the same as in the section above, these values do not directly correspond to one another and are calculated along different dimensional spaces). The kinetic values of $\boldsymbol{D_{eff}}$ and $\boldsymbol{F}$ along each eigenvector of the two component matrices was estimated using traditional maximum likelihood estimation. As a sanity check, we noted that most isolated segments of VAPB diffusion in ER tubules were correctly broken into axes that were parallel or perpendicular to the central cylindrical axis of the ER tubule, and the contribution of diffusion to the perpendicular axis was negligible. Additionally, we noticed that the diffusion of VAPB along the tubule axes simplified to values that are nearly identical to those we have estimated for ER tubule diffusion of another tail anchored protein by a completely unrelated mathematical approach[64].

E. Use of Diffusivity Index to classify latent states

Since we do not constrain the relative contributions of thermal motion and external forces within each segment, the relative contribution of these two terms provides a rough measure of how closely a particular trajectory segment is behaving to unconstrained diffusion. Thus, we introduce a simple "Diffusivity Index" (Extended Data Fig. 4b) for each segment of sufficient length which is defined as the ratio of the thermal term to the force term:

$$i.e., \qquad Diffusivity\ Index \approx \sum_{Traj.Seg.} \frac{\sqrt{2D}}{\gamma^{-1}\vec{\boldsymbol{F}}(\vec{r}_t)}$$

VAPB trajectories in complex ER structures like ER matrices or sheets with two dimensions of freedom show a high Diffusivity Index (though not necessarily a high D_{eff}), while trajectories primarily localized in isolated tubules or thin structures tend to show medium diffusivity indices (yellow and blue, Extended Data Fig. 4c). Trajectories confined to contact sites or immobilized through binding or aggregation show very low diffusivity indices (orange, Extended Data Fig. 4c).

VII. SINGLE CONTACT SITE ANALYSIS AND INTERACTION PROBABILITIES

A. A note on probability distributions and coefficients used throughout this paper

Traditionally, single molecule techniques make use of ensemble-averaged approaches to understand macro-scale phenotypes derived of the sum of components. Where possible, we have tried to use these as well, but as noted above, the high degree of anisotropy and spatial nonuniformity in the system causes a general failure of ergodic conditions even in the non-interacting controls. Thus, we have attempted to create proxies based on simple probabilistic functions throughout the paper to allow readers from biological backgrounds to draw comparison to the fluorescence intensity- and colocalization-based approaches more common in diffraction-limited imaging. We have detailed these additional coefficients and their derivation in the following sections.

B. Localization probabilities and localization density

The spatially-derived probability mass function introduced in the methods serves as the easiest way to identify sites of VAPB interaction in cells, and used to visualize the contact sites in all of the figures of the paper. It is derived by normalizing the localization density (number of localizations per pseudopixel in a defined time window) by the total number of localizations within the calculated space (usually the entire imaging field of view):

$$\rho_{x,y} = \frac{Number\ of\ Localizations\ per\ Computational\ Pixel}{Total\ Number\ of\ Localizations\ in\ Image} \quad \text{such that} \quad \int_{x,y} \rho_{x,y} \equiv 1$$

Thus, the integral of this function over the inside of the refined contact site boundaries provides the relative likelihood that a VAPB molecule selected at random in an arbitrary frame within the window is localized in that specific contact site. These statistics are hard to compare between contact sites in different cells, since they are heavily dependent on the total number of localizations and the number of contact sites in the field of view. However, they can be added across all contact sites of a type (either ERMCSs or other ER CSs) in a single cell to ask about the relative likelihood that a molecule in the cell is at a specific type of contact site in the time window:

$$Probability\ of\ CS\ Localization\ (P_{MitoCS}) = \sum_{i=1}^{N} \int_{MitoCS_{\mathrm{i}}} \rho_{x,y}$$

where N is the number of contact sites in the imaging field that meet the desired criteria. Note that when analyzing VAPB under steady state conditions, the P_{MitoCS} was always significantly less than 0.5 (Extended Data Fig. 2d), reflecting the fact that the vast majority of VAPB is freely diffusing in the ER. This is likely the reason diffraction-limited imaging of VAPB at ERMCSs is so difficult in non-perturbed cells. By contrast, in cells overexpressing PTPIP51, this number can climb as high as 0.9-0.95, indicating the overexpressed tether can recruit much of the VAPB to the expanded contact site when equilibrium is disrupted.

C. Mitochondrial enrichment (ME) coefficient

It is in some situations beneficial to quantify the relative likelihood that a VAPB molecule is at ERMCS as opposed to other (unlabeled) contact sites. For this, we introduce a mitochondrial enrichment coefficient, which is the power of the scaling ratio between the Probability of CS localization for the two contact site subtypes:

$$ME = \log\left(\frac{P_{MitoCS}}{P_{OtherCS}}\right)$$

In this formulation, cells where a tether prefers non-mitochondria sites of interaction will have a negative ME coefficient, and those where tethers prefer mitochondria will have positive ME coefficients. In WT cells under steady-state conditions, cells are fairly uniformly distributed between these two categories (Extended Data Fig. 2e), but under extreme ERMCS expansion conditions, the ME shifts heavily into positive numbers, reflecting the capacity of PTPIP51 to steal potential VAPB tethers from competing partners on other organelles (Fig. 3f).

D. Likelihood of trajectory binding

Related to the localization probability explained in Section 7b above is the likelihood that a single VAPB molecule engages any specific ERMCS. This likelihood is derived by calculating

the proportion of VAPB trajectories that could plausibly bind a contact site in question and measuring the fraction that show direct interaction (see Extended Data Fig. 3a-b):

$$P_{TrajCS} = \frac{Number\ of\ bound\ trajectories}{Total\ number\ of\ trajectories}$$

When calculated over all trajectories in a field of view within one cell, this number is defined to be larger than the P_{MitoCS}, since interacting VAPB molecules at ERMCSs do this for only a short time (and freely diffusing steps do not contribute to the P_{MitoCS} as they do to this coefficient, Extended Data Fig. 3b). We note that when the total number of trajectories considered is restricted to those that physically pass through the space an ERMCS resides, the number increases significantly (Extended Data Fig. 3c, red vs blue), suggesting that while molecules that reach an ERMCS have reasonable chance of interaction, the large expanse of the ER membrane makes these encounters relatively rare. The number generated by this latter analysis is quite variable between contact sites even within a single cell (Extended Data Fig. 3d), suggesting ERMCS-specific regulation may be possible at a local level, and we note it is uncorrelated to the expression level of VAPB (Extended Data Fig. 3e, red dots), supporting the role of the mitochondrial tether in dominating the likelihood of this interaction (Fig. 3).

VIII. A NOTE ON REASONING FOR STATISTICS, ORDER OF OPERATIONS, AND GROUPING OF NESTED DATA

Throughout the paper, we have attempted to choose the appropriate statistical tools such that whenever ambiguity arises, we bias against the tested hypothesis. All data is shown with the median and the 95% confidence interval of the median (except where noted to be otherwise), since several of the datasets throughout the paper were not able to be demonstrated to be clearly drawn from a normal distribution. For datasets that are normal, the median and the mean converge to the same value, so this minimizes the heavy skew that outliers can have on the arithmetic mean for visualization purposes.

All datasets throughout the paper are initially subjected to a series of tests for normality (or lognormality, if possible). Only if all datasets in a family of data to be compared have passed all the tests do we proceed with one-way ANOVA and subsequent Dunn's test for multiple comparisons, in which all conditions are compared to the control. If any of the conditions did not pass the normality tests, then we opted for nonparametic approaches to data analysis with less statistical power. In all cases, the tests used are listed in the captions.

The nature of the contact site data as implemented in this paper is that each contact site is assembled from numerous individual molecular trajectories, many of which interact with numerous different contact sites or show several distinct interactions with the same contact site. As a result, this data is nested over many orders of magnitude (i.e.- experiment day, transfection batch, coverslip, cell, contact site, trajectory, binding interaction, single molecule localization or step). Thus, there are several reasonable ways to group the data, and it is not immediately obvious the most unbiased way to do so. To standardize our approach and avoid confirmation bias from the experimenters in choosing how to group the data, we pooled the data from all the conditions and blindly analyzed which layers of nesting contributed to the largest variation in the data across all the datasets. This was overwhelmingly dominated by the cell-to-cell variation and variation between contact sites within the same cell—which in hindsight is what one would predict for the rapidly tunable system revealed throughout the paper. In no conditions throughout the paper did we find any statistically significant differences between any of the higher levels of nesting, so whenever possible we tried to group the data

at levels of individual cells, reasoning that these represented the best biological replicates for the contact sites within (grouping the data by higher order nests masks the variation in the sample, making the data seem much more homogeneous than it actually is). The few exceptions to this throughout the paper are conditions where the variation between the contact sites was much larger than the variation between cells, so we grouped the data by individual contact site in order to avoid masking the variability in the observations, and this is explicitly stated when this was performed.

Each SPT dataset contains a total of at least 16 regions (20.48 um x 20.48 um) each selected from a different cell divided over at least two experiments. Not all of these contained contact sites (see Extended Data Fig. 2). FIB-SEM data was visually examined in 3 COS7 cells, but all data shown or quantified in the paper comes from a single representative cell. Single contact site analysis was performed on each of the hundreds of contact sites analyzed, each of which contains anywhere from 1-50 VAPB trajectories.

Supplementary References

1. Prinz, W. A., Toulmay, A. & Balla, T. The functional universe of membrane contact sites. *Nat Rev Mol Cell Bio* **21**, 7–24 (2019).

2. Phillips, M. J. & Voeltz, G. K. Structure and function of ER membrane contact sites with other organelles. *Nat Rev Mol Cell Bio* **17**, 69–82 (2016).

3. Pezzati, R., Bossi, M., Podini, P., Meldolesi, J. & Grohovaz, F. High-resolution calcium mapping of the endoplasmic reticulum-Golgi-exocytic membrane system. Electron energy loss imaging analysis of quick frozen-freeze dried PC12 cells. *Mol Biol Cell* **8**, 1501–1512 (1997).

4. Friedman, J. R. *et al.* ER Tubules Mark Sites of Mitochondrial Division. *Science* **334**, 358–362 (2011).

5. Lewis, S. C., Uchiyama, L. F. & Nunnari, J. ER-mitochondria contacts couple mtDNA synthesis with mitochondrial division in human cells. *Science* **353**, aaf5549 (2016).

6. Valm, A. M. *et al.* Applying systems-level spectral imaging and analysis to reveal the organelle interactome. *Nature* **546**, 162–167 (2016).

7. Hoyer, M. J. *et al.* A Novel Class of ER Membrane Proteins Regulates ER-Associated Endosome Fission. *Cell* **175**, 254-265.e14 (2018).

8. Heinrich, L. *et al.* Whole-cell organelle segmentation in volume electron microscopy. *Nature* **599**, 141–146 (2021).

9. Nixon-Abell, J. *et al.* Increased spatiotemporal resolution reveals highly dynamic dense tubular matrices in the peripheral ER. *Science* **354**, aaf3928–aaf3928 (2016).

10. Weigel, A. V. *et al.* ER-to-Golgi protein delivery through an interwoven, tubular network extending from ER. *Cell* **184**, 2412-2429.e16 (2021).

11. Heymann, J. A. W. *et al.* 3D Imaging of mammalian cells with ion-abrasion scanning electron microscopy. *J Struct Biol* **166**, 1–7 (2009).

12. Felts, R. L. *et al.* 3D visualization of HIV transfer at the virological synapse between dendritic cells and T cells. *Proc National Acad Sci* **107**, 13336–13341 (2010).

13. Bennett, A. E. *et al.* Ion-Abrasion Scanning Electron Microscopy Reveals Surface-Connected Tubular Conduits in HIV-Infected Macrophages. *Plos Pathog* **5**, e1000591 (2009).

14. Csordás, G. *et al.* Imaging Interorganelle Contacts and Local Calcium Dynamics at the ER-Mitochondrial Interface. *Mol Cell* **39**, 121–132 (2010).

15. Naon, D. *et al.* Critical reappraisal confirms that Mitofusin 2 is an endoplasmic reticulum–mitochondria tether. *Proc National Acad Sci* **113**, 11249–11254 (2016).

16. Wilson, E. L. & Metzakopian, E. ER-mitochondria contact sites in neurodegeneration: genetic screening approaches to investigate novel disease mechanisms. *Cell Death Differ* **28**, 1804–1821 (2021).

17. Harmon, M., Larkman, P., Hardingham, G., Jackson, M. & Skehel, P. A Bi-fluorescence complementation system to detect associations between the Endoplasmic reticulum and mitochondria. *Sci Rep-uk* **7**, 17467 (2017).

18. Cieri, D. *et al.* SPLICS: a split green fluorescent protein-based contact site sensor for narrow and wide heterotypic organelle juxtaposition. *Cell Death Differ* **25**, 1131–1145 (2018).

19. Lee, J. E., Cathey, P. I., Wu, H., Parker, R. & Voeltz, G. K. Endoplasmic reticulum contact sites regulate the dynamics of membraneless organelles. *Science* **367**, (2020).

20. Giacomello, M. & Pellegrini, L. The coming of age of the mitochondria-ER contact: a matter of thickness. *Cell Death Differ* **23**, 1417–27 (2016).

21. Parlakgül, G. *et al.* Regulation of liver subcellular architecture controls metabolic homeostasis. *Nature* **603**, 736–742 (2022).

22. Hicks, M. L., Brilliant, J. D. & Foreman, D. W. Electron Microscope Comparison of Freeze-Substitution and Conventional Chemical Fixation of Undecalificied Human Dentin. *J Dent Res* **55**, 400–410 (1976).

23. Hoffman, D. P. *et al.* Correlative three-dimensional super-resolution and block-face electron microscopy of whole vitreously frozen cells. *Science* **367**, (2020).

24. Ding, Y. *et al.* Ratiometric biosensors based on dimerization-dependent fluorescent protein exchange. *Nat Methods* **12**, 195–198 (2015).

25. Mattia, T. D. *et al.* FFAT motif phosphorylation controls formation and lipid transfer function of inter-organelle contacts. *Embo J* **39**, e104369 (2020).

26. Stoica, R. *et al.* ALS/FTD-associated FUS activates GSK-3β to disrupt the VAPB–PTPIP51 interaction and ER–mitochondria associations. *Embo Rep* **17**, 1326–1342 (2016).

27. Stoica, R. *et al.* ER–mitochondria associations are regulated by the VAPB–PTPIP51 interaction and are disrupted by ALS/FTD-associated TDP-43. *Nat Commun* **5**, 3996 (2014).

28. De Vos, K. J. *et al.* VAPB interacts with the mitochondrial protein PTPIP51 to regulate calcium homeostasis. *Hum Mol Genet* **21**, 1299–1311 (2012).

29. Csordás, G., Weaver, D. & Hajnóczky, G. Endoplasmic Reticulum–Mitochondrial Contactology: Structure and Signaling Functions. *Trends Cell Biol* **28**, 523–540 (2018).

30. Zung, N. & Schuldiner, M. New horizons in mitochondrial contact site research. *Biol Chem* **401**, 793–809 (2020).

31. Kors, S., Costello, J. L. & Schrader, M. VAP Proteins – From Organelle Tethers to Pathogenic Host Interactors and Their Role in Neuronal Disease. *Frontiers Cell Dev Biology* **10**, 895856 (2022).

32. Csordás, G. *et al.* Structural and functional features and significance of the physical linkage between ER and mitochondria. *J Cell Biology* **174**, 915–921 (2006).

33. Sood, A. *et al.* A Mitofusin-2–dependent inactivating cleavage of Opa1 links changes in mitochondria cristae and ER contacts in the postprandial liver. *Proc National Acad Sci* **111**, 16017–16022 (2014).

34. Anastasia, I. *et al.* Mitochondria-rough-ER contacts in the liver regulate systemic lipid homeostasis. *Cell Reports* **34**, 108873 (2021).

35. Hung, V. *et al.* Proteomic mapping of cytosol-facing outer mitochondrial and ER membranes in living human cells by proximity biotinylation. *Elife* **6**, e24463 (2017).

36. Zheng, P. *et al.* ER proteins decipher the tubulin code to regulate organelle distribution. *Nature* **601**, 132–138 (2022).

37. Murphy, S. E. & Levine, T. P. VAP, a Versatile Access Point for the Endoplasmic Reticulum: Review and analysis of FFAT-like motifs in the VAPome. *Biochim Biophys Acta* **1861**, 952–61 (2016).

38. Kim, Y. J., Guzman-Hernandez, M.-L., Wisniewski, E. & Balla, T. Phosphatidylinositol-Phosphatidic Acid Exchange by Nir2 at ER-PM Contact Sites Maintains Phosphoinositide Signaling Competence. *Dev Cell* **33**, 549–561 (2015).

39. Kirmiz, M., Vierra, N. C., Palacio, S. & Trimmer, J. S. Identification of VAPA and VAPB as Kv2 Channel-Interacting Proteins Defining Endoplasmic Reticulum–Plasma Membrane Junctions in Mammalian Brain Neurons. *J Neurosci* **38**, 7562–7584 (2018).

40. Wu, M. M., Covington, E. D. & Lewis, R. S. Single-molecule analysis of diffusion and trapping of STIM1 and Orai1 at ER-plasma membrane junctions. *Mol Biol Cell* **25**, mbc.E14-06-1107 (2014).

41. Weigel, A. V., Simon, B., Tamkun, M. M. & Krapf, D. Ergodic and nonergodic processes coexist in the plasma membrane as observed by single-molecule tracking. *Proc National Acad Sci* **108**, 6438–6443 (2011).

42. Johnson, B. *et al.* Kv2 potassium channels form endoplasmic reticulum/plasma membrane junctions via interaction with VAPA and VAPB. *Proc National Acad Sci* **115**, E7331–E7340 (2018).

43. Chang, C.-L. & Liou, J. Phosphatidylinositol 4,5-Bisphosphate Homeostasis Regulated by Nir2 and Nir3 Proteins at Endoplasmic Reticulum-Plasma Membrane Junctions. *J Biol Chem* **290**, 14289–14301 (2015).

44. Chang, C.-L., Chen, Y.-J. & Liou, J. ER-plasma membrane junctions: Why and how do we study them? *Biochimica Et Biophysica Acta Bba - Mol Cell Res* **1864**, 1494–1506 (2017).

45. Hsieh, T.-S., Chen, Y.-J., Chang, C.-L., Lee, W.-R. & Liou, J. Cortical actin contributes to spatial organization of ER–PM junctions. *Mol Biol Cell* **28**, 3171–3180 (2017).

46. Field, C. M. & Lénárt, P. Bulk Cytoplasmic Actin and Its Functions in Meiosis and Mitosis. *Curr Biol* **21**, R825–R830 (2011).

47. Schiavon, C. R. *et al.* Actin chromobody imaging reveals sub-organellar actin dynamics. *Nat Methods* **17**, 917–921 (2020).

48. Moore, A. S. *et al.* Actin cables and comet tails organize mitochondrial networks in mitosis. *Nature* **591**, 659–664 (2021).

49. Jaqaman, K. *et al.* Robust single particle tracking in live cell time-lapse sequences. *Nat Methods* **5**, 695–702 (2008).

50. Manley, S. *et al.* High-density mapping of single-molecule trajectories with photoactivated localization microscopy. *Nat Methods* **5**, 155–7 (2008).

51. Freeman, S. A. *et al.* Transmembrane Pickets Connect Cyto- and Pericellular Skeletons Forming Barriers to Receptor Engagement. *Cell* **172**, 305-317.e10 (2018).

52. Kim, S., Leal, S. S., Halevy, D. B., Gomes, C. M. & Lev, S. Structural Requirements for VAP-B Oligomerization and Their Implication in Amyotrophic Lateral Sclerosis-associated VAP-B(P56S) Neurotoxicity. *J Biol Chem* **285**, 13839–13849 (2010).

53. Teuling, E. *et al.* Motor Neuron Disease-Associated Mutant Vesicle-Associated Membrane Protein-Associated Protein (VAP) B Recruits Wild-Type VAPs into Endoplasmic Reticulum-Derived Tubular Aggregates. *J Neurosci* **27**, 9801–9815 (2007).

54. Shi, J., Lua, S., Tong, J. S. & Song, J. Elimination of the Native Structure and Solubility of the hVAPB MSP Domain by the Pro56Ser Mutation That Causes Amyotrophic Lateral Sclerosis. *Biochemistry-us* **49**, 3887–3897 (2010).

55. Borgese, N., Navone, F., Nukina, N. & Yamanaka, T. Mutant VAPB: Culprit or Innocent Bystander of Amyotrophic Lateral Sclerosis? *Contact* **4**, 25152564211022516 (2021).

56. Borgese, N., Iacomino, N., Colombo, S. F. & Navone, F. The Link between VAPB Loss of Function and Amyotrophic Lateral Sclerosis. *Cells* **10**, 1865 (2021).

57. Xu, C. S. *et al.* An open-access volume electron microscopy atlas of whole cells and tissues. *Nature* **599**, 147–151 (2021).

58. Rizzuto, R. *et al.* Close Contacts with the Endoplasmic Reticulum as Determinants of Mitochondrial Ca2+ Responses. *Science* **280**, 1763–1766 (1998).

59. Wang, P. T. C. *et al.* Distinct mechanisms controlling rough and smooth endoplasmic reticulum contacts with mitochondria. *J Cell Sci* **128**, 2759–2765 (2015).

60. Obara, C. J., Moore, A. S. & Lippincott-Schwartz, J. Structural Diversity within the Endoplasmic Reticulum—From the Microscale to the Nanoscale. *Csh Perspect Biol* a041259 (2022) doi:10.1101/cshperspect.a041259.

61. Los, G. V. *et al.* HaloTag: A Novel Protein Labeling Technology for Cell Imaging and Protein Analysis. *Acs Chem Biol* **3**, 373–382 (2008).

62. Encell, L. P. *et al.* Development of a Dehalogenase-Based Protein Fusion Tag Capable of Rapid, Selective and Covalent Attachment to Customizable Ligands. *Curr Chem Genom* **6**, 55–71 (2012).

63. Dong, R. *et al.* Endosome-ER Contacts Control Actin Nucleation and Retromer Function through VAP-Dependent Regulation of PI4P. *Cell* **166**, 408–423 (2016).

64. Sun, Y. *et al.* Unraveling trajectories of diffusive particles on networks. *Phys Rev Res* **4**, 023182 (2022).

65. Calderon, C. P. Motion blur filtering: A statistical approach for extracting confinement forces and diffusivity from a single blurred trajectory. *Phys Rev E* **93**, 053303 (2016).

66. Terasaki, M., Song, J., Wong, J. R., Weiss, M. J. & Chen, L. B. Localization of endoplasmic reticulum in living and glutaraldehyde-fixed cells with fluorescent dyes. *Cell* **38**, 101–108 (1984).

67. Lee, C. & Chen, L. B. Dynamic behavior of endoplasmic reticulum in living cells. *Cell* **54**, 37–46 (1988).

68. Terasaki, M., Chen, L. B. & Fujiwara, K. Microtubules and the endoplasmic reticulum are highly interdependent structures. *J Cell Biology* **103**, 1557–1568 (1986).

69. Lee, C. & Chen, L. B. Endoplasmic Reticulum. *Subcell Biochem* **21**, 343–352 (1993).

70. Terasaki, M. & Reese, T. S. Interactions among endoplasmic reticulum, microtubules, and retrograde movements of the cell surface. *Cell Motil Cytoskel* **29**, 291–300 (1994).

71. Waterman-Storer, C. M. & Salmon, E. D. Endoplasmic reticulum membrane tubules are distributed by microtubules in living cells using three distinct mechanisms. *Curr Biol* **8**, 798–807 (1998).

72. Waterman-Storer, C. M., Gregory, J., Parsons, S. F. & Salmon, E. D. Membrane/microtubule tip attachment complexes (TACs) allow the assembly dynamics of plus ends to push and pull membranes into tubulovesicular networks in interphase Xenopus egg extracts. *J Cell Biology* **130**, 1161–1169 (1995).

73. Nehls, S. *et al.* Dynamics and retention of misfolded proteins in native ER membranes. *Nat Cell Biol* **2**, 288–295 (2000).

74. York, A. G. *et al.* Instant super-resolution imaging in live cells and embryos via analog image processing. *Nat Methods* **10**, 1122–1126 (2013).

75. Li, D. *et al.* ADVANCED IMAGING. Extended-resolution structured illumination imaging of endocytic and cytoskeletal dynamics. *Sci New York N Y* **349**, aab3500 (2015).

76. Guo, Y. *et al.* Visualizing Intracellular Organelle and Cytoskeletal Interactions at Nanoscale Resolution on Millisecond Timescales. *Cell* **175**, 1430-1442.e17 (2018).

77. Chen, S., Novick, P. & Ferro-Novick, S. ER network formation requires a balance of the dynamin-like GTPase Sey1p and the Lunapark family member Lnp1p. *Nat Cell Biol* **14**, 707–716 (2012).

78. Hu, J. *et al.* A Class of Dynamin-like GTPases Involved in the Generation of the Tubular ER Network. *Cell* **138**, 549–561 (2009).

79. Renvoisé, B. *et al.* Reep1 null mice reveal a converging role for hereditary spastic paraplegia proteins in lipid droplet regulation. *Hum Mol Genet* **25**, 5111–5125 (2016).

80. Chang, J., Lee, S. & Blackstone, C. Spastic paraplegia proteins spastizin and spatacsin mediate autophagic lysosome reformation. *J Clin Investigation* **124**, 5249–62 (2014).

81. Liu, T. Y. *et al.* Cis and trans interactions between atlastin molecules during membrane fusion. *Proc National Acad Sci* **112**, E1851–E1860 (2015).

82. Wang, S., Tukachinsky, H., Romano, F. B. & Rapoport, T. A. Cooperation of the ER-shaping proteins atlastin, lunapark, and reticulons to generate a tubular membrane network. *Elife* **5**, e18605 (2016).

83. Shen, H. *et al.* Single Particle Tracking: From Theory to Biophysical Applications. *Chem Rev* **117**, 7331–7376 (2017).

84. Diezmann, A. von, Shechtman, Y. & Moerner, W. E. Three-Dimensional Localization of Single Molecules for Super-Resolution Imaging and Single-Particle Tracking. *Chem Rev* **117**, 7244–7275 (2017).

85. Manzo, C. & Garcia-Parajo, M. F. A review of progress in single particle tracking: from methods to biophysical insights. *Rep Prog Phys* **78**, 124601 (2015).

86. Chenouard, N. *et al.* Objective comparison of particle tracking methods. *Nat Methods* **11**, 281–289 (2014).

87. Beheiry, M. E. *et al.* A Primer on the Bayesian Approach to High-Density Single-Molecule Trajectories Analysis. *Biophys J* **110**, 1209–15 (2016).

88. Masson, J.-B. *et al.* Mapping the energy and diffusion landscapes of membrane proteins at the cell surface using high-density single-molecule imaging and Bayesian inference: application to the multiscale dynamics of glycine receptors in the neuronal membrane. *Biophys J* **106**, 74–83 (2014).

89. Parutto, P., Heck, J., Heine, M. & Holcman, D. Biophysics of high density nanometer regions extracted from super-resolution single particle trajectories: application to voltage-gated calcium channels and phospholipids. *Sci Rep-uk* **9**, 18818 (2019).

90. Hua, R. *et al.* VAPs and ACBD5 tether peroxisomes to the ER for peroxisome maintenance and lipid homeostasis. *J Cell Biol* **216**, 367–377 (2017).

91. Prosser, D. C., Tran, D., Gougeon, P.-Y., Verly, C. & Ngsee, J. K. FFAT rescues VAPA-mediated inhibition of ER-to-Golgi transport and VAPB-mediated ER aggregation. *J Cell Sci* **121**, 3052–3061 (2008).

92. Suzuki, H. *et al.* ALS-linked P56S-VAPB, an aggregated loss-of-function mutant of VAPB, predisposes motor neurons to ER stress-related death by inducing aggregation of co-expressed wild-type VAPB: Detailed characterization of VAPB/ALS8. *J Neurochem* **108**, n/a-n/a (2010).

93. Yamanaka, T., Nishiyama, R., Shimogori, T. & Nukina, N. Proteomics-Based Approach Identifies Altered ER Domain Properties by ALS-Linked VAPB Mutation. *Sci Rep-uk* **10**, 7610 (2020).

94. Papiani, G. *et al.* Restructured endoplasmic reticulum generated by mutant amyotrophic lateral sclerosis-linked VAPB is cleared by the proteasome. *J Cell Sci* **125**, 3601–3611 (2012).

95. Calderon, C. P., Weiss, L. E. & Moerner, W. E. Robust hypothesis tests for detecting statistical evidence of two-dimensional and three-dimensional interactions in single-molecule measurements. *Phys Rev E* **89**, 052705 (2014).

96. Persson, F., Lindén, M., Unoson, C. & Elf, J. Extracting intracellular diffusive states and transition rates from single-molecule tracking data. *Nat Methods* **10**, 265–269 (2013).

97. Monnier, N. *et al.* Inferring transient particle transport dynamics in live cells. *Nat Methods* **12**, 838–840 (2015).

98. Schwantes, C. R., McGibbon, R. T. & Pande, V. S. Perspective: Markov models for long-timescale biomolecular dynamics. *J Chem Phys* **141**, 090901 (2014).

99. Grimm, J. B. *et al.* A general method to improve fluorophores for live-cell and single-molecule microscopy. *Nat Methods* **12**, 244–50, 3 p following 250 (2015).

100. Grimm, J. B. *et al.* Bright photoactivatable fluorophores for single-molecule imaging. *Nat Methods* **13**, 985–988 (2016).

101. Grimm, J. B. *et al.* A general method to fine-tune fluorophores for live-cell and in vivo imaging. *Nat Methods* **14**, 987–994 (2017).

102. Calderon, C. Data-Driven Techniques for Detecting Dynamical State Changes in Noisily Measured 3D Single-Molecule Trajectories. *Molecules* **19**, 18381–18398 (2014).

103. Calderon, C. P. & Bloom, K. Inferring Latent States and Refining Force Estimates via Hierarchical Dirichlet Process Modeling in Single Particle Tracking Experiments. *Plos One* **10**, e0137633 (2015).

104. Giamogante, F., Barazzuol, L., Brini, M. & Calì, T. ER–Mitochondria Contact Sites Reporters: Strengths and Weaknesses of the Available Approaches. *Int J Mol Sci* **21**, 8157 (2020).
