# Supplementary material for: Motion of VAPB molecules reveals ER–mitochondria contact site subdomains
Source: Nature. 2024 Jan 24;626(7997):169–76. doi: 10.1038/s41586-023-06956-y (PMC10830423; doi:10.1038/s41586-023-06956-y)
Supplement: Supplementary file 2 — Reporting Summary [file 41586_2023_6956_MOESM2_ESM.pdf]

nature portfolio

Corresponding author(s): Christopher Obara
Jennifer Lippincott-Schwartz

Last updated by author(s): 11/27/2023

Reporting Summary

Nature Portfolio wishes to improve the reproducibility of the work that we publish. This form provides structure for consistency and transparency in reporting. For further information on Nature Portfolio policies, see our Editorial Policies and the Editorial Policy Checklist.

Statistics

For all statistical analyses, confirm that the following items are present in the figure legend, table legend, main text, or Methods section.

n/a	Confirmed	
☐	☒	The exact sample size (*n*) for each experimental group/condition, given as a discrete number and unit of measurement
☐	☒	A statement on whether measurements were taken from distinct samples or whether the same sample was measured repeatedly
☐	☒	The statistical test(s) used AND whether they are one- or two-sided<br>*Only common tests should be described solely by name; describe more complex techniques in the Methods section.*
☐	☒	A description of all covariates tested
☐	☒	A description of any assumptions or corrections, such as tests of normality and adjustment for multiple comparisons
☐	☒	A full description of the statistical parameters including central tendency (e.g. means) or other basic estimates (e.g. regression coefficient) AND variation (e.g. standard deviation) or associated estimates of uncertainty (e.g. confidence intervals)
☐	☒	For null hypothesis testing, the test statistic (e.g. *F*, *t*, *r*) with confidence intervals, effect sizes, degrees of freedom and *P* value noted<br>*Give P values as exact values whenever suitable.*
☐	☒	For Bayesian analysis, information on the choice of priors and Markov chain Monte Carlo settings
☐	☒	For hierarchical and complex designs, identification of the appropriate level for tests and full reporting of outcomes
☒	☐	Estimates of effect sizes (e.g. Cohen's *d*, Pearson's *r*), indicating how they were calculated

Our web collection on statistics for biologists contains articles on many of the points above.

Software and code

Policy information about availability of computer code

Data collection	Nikon Elements AR 6.0, ZEN Black 2.3<br>The microscopes are operated using standard commercial software from Nikon (Nikon Elements) or Zeiss (ZEN Black), but can in principle be collected using any custom or public operation software for optical hardware.
Data analysis	Fiji-ImageJ (v.1.54f) with TrackMate plugin (v7.11.1)<br>Matlab 2022a (2015b for Ursa Analytics Code)<br>Python (v. 2.7.13)<br>All code used throughout manuscript is freely available at Github through the following repository (and associated linked repositories): https://github.com/cjobara/Obara-Nixon-Abell-2023-Nature

For manuscripts utilizing custom algorithms or software that are central to the research but not yet described in published literature, software must be made available to editors and reviewers. We strongly encourage code deposition in a community repository (e.g. GitHub). See the Nature Portfolio guidelines for submitting code & software for further information.

Data

Policy information about availability of data

All manuscripts must include a data availability statement. This statement should provide the following information, where applicable:

- Accession codes, unique identifiers, or web links for publicly available datasets
- A description of any restrictions on data availability
- For clinical datasets or third party data, please ensure that the statement adheres to our policy

The experimental data used in this paper are freely available in both raw and processed form at Figshare with the identifier: https://doi.org/10.25378/janelia.c.6916543.v1. All data are additionally available from the authors in other formats as needed upon request.

Research involving human participants, their data, or biological material

Policy information about studies with human participants or human data. See also policy information about sex, gender (identity/presentation), and sexual orientation and race, ethnicity and racism.

Reporting on sex and gender	N/A - There is no data of this type in the paper.
Reporting on race, ethnicity, or other socially relevant groupings	N/A - There is no data of this type in the paper.
Population characteristics	N/A - There is no data of this type in the paper.
Recruitment	N/A - There is no data of this type in the paper.
Ethics oversight	N/A - There is no data of this type in the paper.

Note that full information on the approval of the study protocol must also be provided in the manuscript.

Field-specific reporting

Please select the one below that is the best fit for your research. If you are not sure, read the appropriate sections before making your selection.

☒ Life sciences ☐ Behavioural & social sciences ☐ Ecological, evolutionary & environmental sciences

For a reference copy of the document with all sections, see nature.com/documents/nr-reporting-summary-flat.pdf

Life sciences study design

All studies must disclose on these points even when the disclosure is negative.

Sample size	Each SPT dataset contains a total of at least 16 regions (20.48 um x 20.48 um) each selected from a different cell divided over at least two experiments. Not all of these contained contact sites (see Extended Data Fig. 2). FIB-SEM data was visually examined in 3 COS7 cells and 1 U2-OS cell, but all data shown or quantified in the paper comes from a single representative COS7 cell. Single contact site analysis was performed on each of the hundreds of contact sites analyzed, each of which contains anywhere from 1-50 VAPB trajectories. No statistics were performed to determine appropriate sample size, and sample size per condition was largely governed by the technical limitations of the experiment and how many cells could be collected within the time window when the sample was healthy (see Supplemental Text, Sections IVA and VA for detailed explanation). All of the samples imaged showed variability across the entire range of possible values in the specific locations and functions of tethers, even over short time scales (see Ext. Data Fig. 2), and this was consistent in every independent experiment run. Thus, we feel the samples presented are sufficient to demonstrate the variation of the system, which is our major conclusion.
Data exclusions	No data was excluded from this study, though density-based identification of contact sites is performed manually based on the VAPB expression level in the cell. Thus, contact sites that do not have sufficient VAPB molecule interactions during the movie are likely removed from the dataset by lack of recognition.
Replication	All experiments in the paper were performed at least two independent times. No significant differences were observed between independent experiments--all samples examined showed consistent dynamic exchange and cell-to-cell variability across the range of potential tethering behaviors (see Extended Data Fig. 2 for quantification). The FIB-SEM volume presented contains two COS7 cells, and we visually checked the contact sites were similar in shape and abundance in both cells an additional two datasets that belong to other experimenters (one COS7 cell and one U2-OS cell). On account of the labor required, we only performed full reconstructions in the two subvolumes used in the paper, which come from the same cell (and a single control volume that is provided in the data repository but not the paper, since it did not have specific contact sites). All this data is provided in the Figshare repository.
Randomization	We did not randomize any aspects of the study, save the order in which datasets had manual thresholding applied. This was done in a random order to avoid bias about how thresholds were applied.
Blinding	SPT experiments are always performed essentially blind, since the experimenter cannot see the data while setting up the scope and initiating

Blinding	the imaging acquisition. Regions that are flat enough to perform SPT are selected using the structure of the ER (which is visible to the experimentalist). All downstream analysis is performed with the analyst blinded and the data in a scrambled order, thus, all priors and parameters are established blind. However, the majority of this pipeline is automated regardless, as such, there is little space for the analyst to affect the results even if they knew which dataset they worked with.

Reporting for specific materials, systems and methods

We require information from authors about some types of materials, experimental systems and methods used in many studies. Here, indicate whether each material, system or method listed is relevant to your study. If you are not sure if a list item applies to your research, read the appropriate section before selecting a response.

Materials & experimental systems

n/a	Involved in the study	
☒	☐	Antibodies
☐	☒	Eukaryotic cell lines
☒	☐	Palaeontology and archaeology
☒	☐	Animals and other organisms
☒	☐	Clinical data
☒	☐	Dual use research of concern
☒	☐	Plants

Methods

n/a	Involved in the study	
☒	☐	ChIP-seq
☒	☐	Flow cytometry
☒	☐	MRI-based neuroimaging

Eukaryotic cell lines

Policy information about cell lines and Sex and Gender in Research

Cell line source(s)	All experiments in the paper are performed using COS7 cells from ATCC, experiments are performed within 40 passages of the initial shock provided.
Authentication	We have not performed any authentication of the lines beyond purchasing a second aliquot from ATCC and performing an independent repeat of some of the SPT experiments in them. There were no obvious differences in any property examined.
Mycoplasma contamination	Cells were all free of mycoplasma at the time of experimentation, and are tested routinely during passaging.
Commonly misidentified lines (See ICLAC register)	No commonly misidentified cell lines were used in this study.
